# Wavelet-based algorithm to the evaluation of contrasted hepatocellular carcinoma in CT-images after transarterial chemoembolization

**DOI:** 10.1186/1748-717X-9-166

**Published:** 2014-07-26

**Authors:** Matheus Alvarez, Diana Rodrigues de Pina, Fernando Gomes Romeiro, Sérgio Barbosa Duarte, José Ricardo de Arruda Miranda

**Affiliations:** 1 Instituto de Biociências de Botucatu, Departamento de Física e Biofísica, UNESP - Universidade Estadual Paulista, Distrito de Rubião Junior S/N, Botucatu, 18618-000 São Paulo, Brazil; 2 Faculdade de Medicina de Botucatu, Departamento de Doenças Tropicais e Diagnóstico por Imagem, UNESP - Universidade Estadual Paulista, Distrito de Rubião Junior S/N, Botucatu, 18618-000 São Paulo, Brazil; 3 Faculdade de Medicina de Botucatu, Departamento de Clínica Médica, UNESP - Universidade Estadual Paulista, Distrito de Rubião Junior S/N, Botucatu, 18618-000 São Paulo, Brazil; 4 Centro Brasileiro de Pesquisas Físicas- CBPF/MCT, Rio de Janeiro, 22290-180 Rio de Janeiro, Brazil

## Abstract

**Background:**

Hepatocellular carcinoma is a primary tumor of the liver and involves different treatment modalities according to the tumor stage. After local therapies, the tumor evaluation is based on the mRECIST criteria, which involves the measurement of the maximum diameter of the viable lesion. This paper describes a computed methodology to measure through the contrasted area of the lesions the maximum diameter of the tumor by a computational algorithm.

**Methods:**

63 computed tomography (CT) slices from 23 patients were assessed. Non-contrasted liver and HCC typical nodules were evaluated, and a virtual phantom was developed for this purpose. Optimization of the algorithm detection and quantification was made using the virtual phantom. After that, we compared the algorithm findings of maximum diameter of the target lesions against radiologist measures.

**Results:**

Computed results of the maximum diameter are in good agreement with the results obtained by radiologist evaluation, indicating that the algorithm was able to detect properly the tumor limits. A comparison of the estimated maximum diameter by radiologist versus the algorithm revealed differences on the order of 0.25 cm for large-sized tumors (diameter > 5 cm), whereas agreement lesser than 1.0 cm was found for small-sized tumors.

**Conclusions:**

Differences between algorithm and radiologist measures were accurate for small-sized tumors with a trend to a small decrease for tumors greater than 5 cm. Therefore, traditional methods for measuring lesion diameter should be complemented non-subjective measurement methods, which would allow a more correct evaluation of the contrast-enhanced areas of HCC according to the mRECIST criteria.

## Background

Hepatocellular carcinoma (HCC) is the fifth most common cancer in men and the seventh most common in women [1]. Its incidence is highest in regions where hepatitis B virus is endemic [1]. In the United States, deaths caused by hepatitis C virus (HCV)-related HCC are rapidly rising. In the past two decades, the incidence of HCC in the United States has tripled, but the 5-year survival rate has remained below 12% [2]. The greatest proportional increase in cases of HCC has been seen among Hispanics and whites between 45 and 60 years old [2].

In general, HCC diagnosis is based on noninvasive imaging tests [3,4]. In patients with cirrhosis and a focal hepatic lesion ≥ 2 cm, the diagnosis may be confidently established on the basis of typical imaging features showing areas of arterial enhancement and regions promptly “washed out” (fainter than the liver tissue) in the venous or delayed phase of four-phase multidetector computed tomography (CT) exam (where the four phases are unenhanced, arterial, venous, and delayed) [3,5].

Orthotopic liver transplantation (OLT) is the recommended treatment when the tumor is within specific criteria, that based on the tumor maximum diameters and the absence of tumor spread outside the liver [3]. Previous studies support the effectiveness of OLT in patients meeting the Milan criteria adopted by the United Network for Organ Sharing (UNOS) [4,5]. The Milan criteria states that orthotopic liver transplantation is recommended only if the patient have a solitary HCC nodule with a diameter ≤ 5 cm or no more than 3 nodules with diameters ≤ 3 cm [5,6].

When any kind of local therapy is used in order to reduce the tumor size, the HCC nodules are evaluated according to the mRECIST criteria, which require that only well-delineated, arterially enhanced lesions could be selected as viable tumor tissue [7]. Using Computed Tomography (CT) images, the viable tumor is measured in the arterial phase, with highest distinction between the viable vascularized tumor nodule and necrotic tisue (non enhanced region) [8-11]. The longest diameter of the viable tumor should be carefully assessed on the CT examination, since it can change the decision of which treatment will be further indicated to each patient [7]. The measurement of the viable tumor diameter should not include any major intervening areas of necrosis [7]. However, large nodules and/or tumors previously submitted to local therapies are often filled by necrotic areas, making difficult to calculate the viable area of these tumors [8].

Before a curative treatment, many patients with HCC receive non-curative therapies in order to achieve the best survival rates, and one of the most used of them is the transarterial chemoembolization (TACE). This kind of treatment consists of infusions of a chemotherapeutic agent through the vessel that is the responsible by the tumor nutrition [9]. As soon the infusion is finished, the vessel is closed in order to isolate the chemotherapeutic agent within the tumor [9]. As a result, it causes a local destruction of the initial lesion, which can be seen as a necrotic area within the tumor.

The TACE efficacy can be measured by the decrease of the target lesion, and when the tumor has enough reduction. The patients can be considered at low disease stage, allowing them to receive OLT as a curative treatment [6]. However, there are some difficult to distinguish between patients which are good candidates to OLT and those still needing to receive other sessions of TACE or another therapy, taking only into account a single measure of the maximum tumor diameter. Other criterion, as the WHO criterion is based on the sum of bidimensional perpendicular products. Another one is the RECIST criterion which is based on the unidimesional quantity, the sum of the longest found diameters. Both criteria were designed for the evaluation of cytotoxic agents and not for local therapies. TACE induces tumor necrosis with or without changes in tumor size [12]. Because of this, the WHO and RECIST criteria have been considered as suboptimal methods for tumor response assessment in HCC patients undergoing TACE. As a result, the European Association for the Study of the Liver (EASL) and the American Association for the Study of Liver Disease (AASLD) have proposed new methods, including the concept of viable enhancing lesion modifying WHO (EASL) and RECIST (mRECIST) criteria, respectively [8]. The EASL and mRECIST criteria resulted in a higher objective response rate and provided more reliable prognostic information, including survival, than conventional WHO and RECIST criteria. So, the measurements of the viable areas of HCC nodules are now the best way to access the TACE efficiency [9].

In this work, we measured the maximum diameter of the viable HCC by a computed algorithm in patient after TACE. For this purpose, an algorithm based on the discrete wavelet transform (DWT) was developed to quantify the enhanced areas of the liver on CT images using a non subjective way.

## Methods

### CT data sample

The CT data for this study comprises 23 enhanced high-resolution abdomen CT exams offered by the Clinical Hospital of Botucatu Medical School. Ethical approval was granted by Research Ethical Commission of this institute under the protocol number 485/2012. Written informed consent was obtained from the patient for the publication of this report and any accompanying images. These exams included CT scans performed after transarterial chemoembolization of 23 patients who had not been treated by another kind of therapy. The hospital radiologist selected 63 slices which contains at least one contrasted part of a typical HCC lesion. There were selected the slices that had the highest amount of tumoral contrast-enhanced tissue. Each CT image measured 512 × 512 pixels, the pixel size ranged from 0.73 to 0.89 mm, with mean pixel value 0.80 mm. The HCC maximum contrasted diameter size evaluated by an expert radiologist, with more than three years of experience, varies from 1.4 cm to 13.3 cm, central value of 4.7 cm and mean ± standarddeviation of 5.3 ± 3.0 cm.

### Patients

We included patients undergoing 4-phases tomography at Botucatu Medical School, who have all of the following conditions: (i) more than 18 years old; (ii) undoubted diagnosis of cirrhotic liver and HCC superior to 1 cm of diameter; (iii) at least one contrast-enhanced lesion at the arterial phase; (iv) rapid washout of the lesion during the venous phase; (v) without previous lipiodol use in HCC lesions treated through transarterial chemoembolization.

### Highlighting structures using wavelets

Multiscale contrast enhancement was used to highlight the tumor inside the liver. Similar to Fourier analysis, the WT corresponds to a decomposition of the functional representation of the digital image. Whereas Fourier transformation represents the signal in the frequency domain, the WT provides a spatio-frequency decomposition of the signal [10,11].

This section describes image decomposition by multiresolution analysis (MRA), which has the ability to separate the decomposition into higher frequency bands and residuals (low-frequency components). This method offers multiresolution properties and highlights the characteristics of interest in the image. By introducing a high band-pass function *ψ* and a low-pass scaling function *φ*, a one-dimensional (1D) signal *f*(*x*) can be decomposed by MRA as:

(1) $$f\left(x\right)=\sum_{k}\sum_{j=1}^{J}d_{j}\left(k\right)\cdot \psi_{j,k}\left(x\right)+\sum_{k}C_{J}\left(k\right)\cdot \varphi_{J,k}\left(x\right)$$

where the first and second terms on the right-hand side of equation (1) represent the decomposed high- and low-frequency components of the image, respectively [13]. The wavelet coefficients *d*_*j*_(*k*) are given by the scalar products of the original image with the *ψ*_*j*,*k*_ basis elements in the pixel position *k* (covering the row image) and the chosen decomposition level *j. C*_*J*_(*k*) are the scaling coefficients, and *φ*_*J*,*k*_(*x*) are the respective scaling functions [12].

In the case of an image *f*(*x*, *y*), for *j* = 1, 1D high-/low-pass filtering is first applied to the original image *f*(*x*, *y*) along the horizontal direction (0°), followed by a decimation in which every odd-numbered element is removed. The 1D filtering and decimation are then applied in the vertical direction (90°). According to the combination (high-high, high-low, low-high, and low-low filtering), the output consists of four quadrants (*q*). The lowest-resolution quadrant corresponds to the sub-bands for the scaling coefficient *C*_1_(*k*). The other three quadrants with directionalities {0°, 90°, 45°} are the sub-bands for the wavelet coefficients *d*_1_(*k*, *q*). The process is repeated for *j* = 2 by using *C*_1_(*k*) in place of the original image [13].

The coefficients *d*_j_ (*k*, *q*) indicate the high-frequency elements of the decomposed image at position *k* and quadrant *q* within the frequency band *j*, where *j* is usually referred to as the decomposition/ resolution level (or simply as the “level”). As the level *j* increases, the structural information of the image object in *d*_*j*_ (*k*, *q*) decreases [13]. Therefore, the wavelet coefficients used for the discrete WT may be chosen according to the size of the structure that one wants to highlight in the image. Alternatively, the images may be reconstructed to improve algorithm performance via MRA [14-16].

### Algorithm optimization method

**Figure 1:**
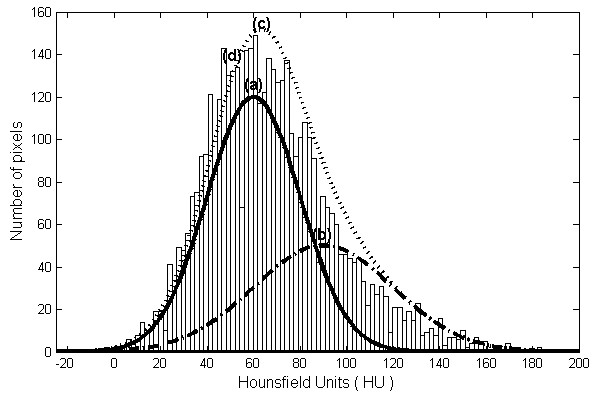
**Distributions of pixel intensity for liver tissues.** Distributions of pixel intensity for a real image containing normal curve-(a) and contrasted liver tissue curve-(b). the Gaussian distributions present mean value and dispersions 55 ± 10 HU for normal tissue and 90 ± 11 HU for contrasted liver tissue. The superposition of (a) and (b) distribution is represented by the curve (c), and (d) is actual histogram extracted from the image.

**Figure 2:**
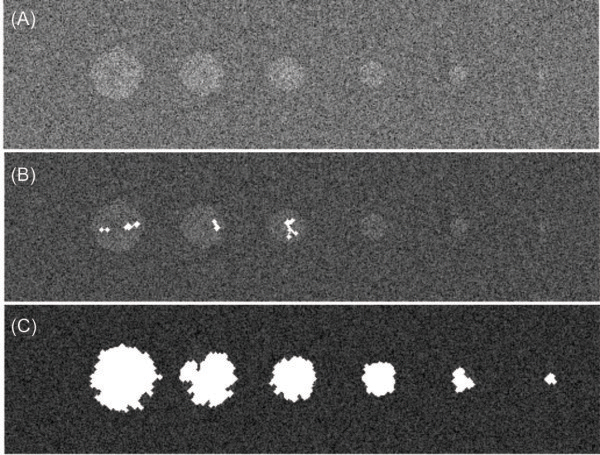
**Virtual phantom and algorithm results.** Virtual phantom constituted by simulated liver tissue and encrusted carcinomas (circles with diameters of 10 cm, 8 cm, 6 cm, 4 cm, 2 cm and 0.5 cm) in part **(A)**. This phantom was used to optimize the algorithm performance. In **(B)** an illustrative example of the algorithm performance without wavelet filtering and in **(C)** an illustration of the algorithm performance to highlight the HCC simulated area.

**Figure 3:**
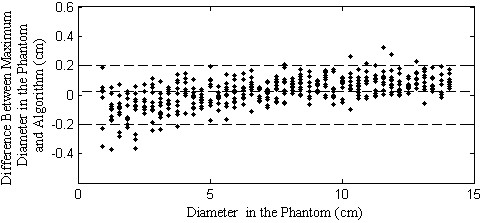
**Algorithm results when applied to the phantom.** Scatter plot of differences between algorithm measure of maximum diameter of the masses of the phantom (Figure 2), an agreement of ±0.2 cm is clearly shown. The central line corresponds to the mean value of deviations. The upper and lower lines depict the limits of 2 SDs.

Pixel intensities (in Hounsfield units, HU) of each slice were studied using MatLab® platform. The gray intensity levels of the pixels in regions containing enhanced and normal liver tissues were analyzed. The pixel intensity distribution in each type of tissue was fitted by Gaussians and the mean and SD determined in the slices was determined, as shown in Figure 1. The curve for normal liver tissue is depicted in part (a), contrasted liver tissue distributions in part (b), the superposition of (a) and (b) distribution is represented by the curve (c), and (d) is actual histogram extracted from the image.A virtual phantom was developed for the algorithm optimization. The phantom was used to optimize the detection of HCC and remarking the differences from normal liver tissue as shown in the phantom image of Figure 2(A).When constructing the phantom, the Gaussian distribution representing the normal tissue was used to fill a 256 × 512 -pixel field image as a background. The pixels intensities for this environment were simulated according to the distribution represented by curve-(a) in Figure 1. A set of HCC lesions were simulated by cycles with maximum diameter from 5 mm to 100 mm incrusted to that background. The circle areas were filled with pixels with intensity pseudorandomly generated by the Gaussian curve (b) in Figure 1. Several algorithms and wavelets filters for segmenting and quantifying the image structures were used until to get the best results. The final algorithm and filter configuration are described in next section.When calculating the efficiency of the algorithm, the diameters of the created circles in the liver were compared with the diameter measured by the algorithm. Circles of maximum diameters varying from 0.5 cm to 14.0 cm, in steps of 0.5 cm and 10 iterations each size were generated and used as input to the algorithm. This comparison is shown as a scatter plot located at Figure 3. Bland-Altman Limits of Agreement (LoA) encountered were in the range of -0.32 cm and 0.31 cm and an R squared equals to 0.99 for a linear fit, which is an acceptable limit of agreement.

### Optimized algorithm description

The algorithm reads the DICOM image of the slice of interest, and the operator makes a manual segmentation of the liver tissue. For this step, a non automatic process is used, because the set of analyzed CT were previously segmented in the routine service. In fact, there are several algorithms available that can semi automatically segment the liver tissue with good results [17]. Of note, HCC is highly related to other liver diseases (i.e., hepatitis, venous thrombosis, cirrhosis) that may deform the liver structure. Being so, we remark that manual segmentation of the liver tissue avoids the quantification of unwanted structures.

The discrete WT frequency bands were estimated in 1D by using equation (2) [18]:

(2) $$\xi_{s}\left(\psi \right)=\frac{\xi_{0}\left(\psi \right)}{2^{s}p}$$

where *ξ*_*s*_(*ψ*) is the center spatial frequency (mm^-1^) in the scale *s*, *p* is the pixel pitch, and *ξ*_0_(*ψ*) is the pseudo-frequency (mm^-1^) of the wavelet in its basic level (*ψ*_*o*,*o*_). In this study, Daubechies 10 (*ξ*_0_(*ψ*) ≅ 0.693) [16,19] was used as the mother wavelet in all of the procedures. This asymmetric, orthogonal wavelet has many image-processing applications and shows better qualitative results than wavelets from other families [20-27]. The frequency bands chosen to reconstruct the ROI images were determined by considering the size of the HCC lesion. Approximation coefficients above level 2 (≅ 0.22 mm) were used to reconstruct all of the images, because such coefficients remove small-sized fluctuations (but not small-sized tumors) from the image.

The output of the previous step was binarized with a threshold of M_NORMAL_ + STD_NORMAL_, as stated in equation (3),

(3) $$B_{\mathrm{output}}^{1}\left(x,y\right)=\{\begin{array}{cc} 1 & \mathrm{if}\;I\left(x,y\right)>M_{\mathrm{NORMAL}}\\ 0 & \mathrm{else} \end{array}$$

where *I* (*x*, *y*) is the input image. After binarization, erosion (4) and dilation (5) filters were applied in the image $B_{\mathrm{output}}^{1}\left(x,y\right)$:

(4) $$B_{\mathrm{output}}^{2}\left(x,y\right)=\{\begin{array}{cc} 0 & \mathrm{if}\;B_{\mathrm{output}}^{1}\left(x+i-2,y+j-2\right)=1\;\mathrm{for}\;\mathrm{all}\;i,j=1,2,3\\ 1 & \mathrm{else} \end{array}$$

(5) $$B_{\mathrm{output}}^{3}\left(x,y\right)=\{\begin{array}{cc} 1 & \mathrm{if}\;B_{\mathrm{output}}^{2}\left(x+i-2,y+j-2\right)=1\;\mathrm{for}\;\mathrm{all}\;i,j=1,2,3\\ 0 & \mathrm{else} \end{array}$$

In these equations, *i* and *j* are arbitrary labels for the neighbors of the pixel being analyzed. Erosion and dilation filters (when applied sequentially in an image) have the property of smoothing the objects in the binary image and removing small-sized objects produced by binarization [15,28].

### Statistical assessment

Three equivalent groups to the measured diameter by the radiologist were used: (i) The G1, for which nodules equal or less than 3 cm in HCC diameter; (ii) The G2 with nodules between 3 and 5 cm in HCC diameter and (iii) The G3, where HCC diameters were above 5 cm. A group of three experienced radiologists, with more than 5 years of experience each, scored the images together agreeing with each other in the measure of the maximum diameter of the lesion. The separation criteria were based on the limits stipulated by Barcelona Clinic Liver Cancer (BCLC) staging, in which multinodular lesions greater than 3.0 cm or unique lesions greater than 5.0 cm are intermediate HCC and cannot be submitted to liver transplantation before tumor reduction [3]. Dependency was evaluated by the R-square produced by a linear fitting of computed against radiologist measures. T-student test was applied to conclude if there is statistical difference between the groups of measures and Bland-Altmann plots were also used to assess the dispersion and limits of agreement between measures [29].

## Results and discussion

**Figure 4:**
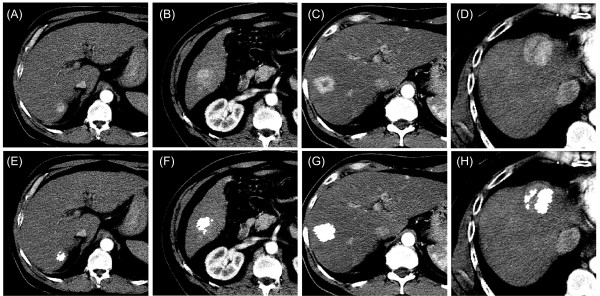
**Highlighting of enhanced HCC in real CT-images.** Input images **(A-D)** with the correspondent output of the algorithm **(E-H)** in the same column. CT slices were obtained from exams of 4 different patients with HCCs, showing the detected HCCs (highlighted region) in the output images.

The optimized algorithm was used to detect HCC in the liver of actual CT images. Figure 4 shows some examples of the algorithm performance for highlighting the HCC region. The algorithm located and dimensioned the lesion in all images with high precision. The radiologist opinion was in quite good agreement with the delimitation region of the tumor for all the data set.

**Figure 5:**
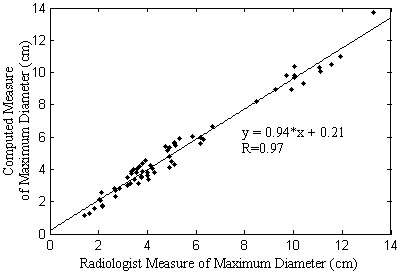
**Radiologist and algorithm correlation in measures.** Comparison between the maximum diameters determined by radiologist and by the algorithm. Diameter evaluated by radiologist versus diameter evaluated by the algorithm linear fit obtained an R-square of 0.97 and t-student test presented a *p* of 0.13, not indicating difference significant.

**Figure 6:**
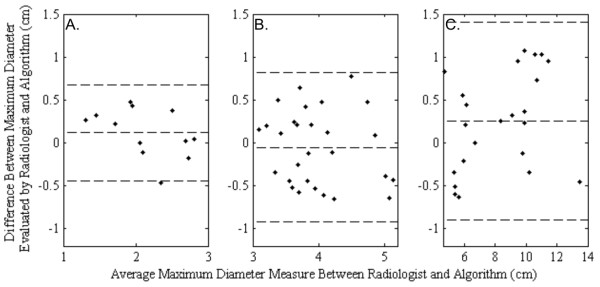
**Algorithm bearing for small, medium and large HCC enhancement.** Bland-Altmann plots of the nodules equal or less than 3 cm in HCC diameter **(A)**, nodules between 3 and 5 cm in HCC diameter **(B)** diameters above 5 cm measured by the radiologist **(C)**. The central lines corresponds to the mean value of deviations. The upper and lower lines depict the limits of 2 SDs.

Validation of the algorithm results was carried out by comparing measured diameters using the algorithm with the radiologist evaluations. Data were divided in three groups according to the maximum diameters calculated by the radiologist, as stated in the statistical assessment session. Figure 5 compares the results obtained from the algorithm computed measures with the results evaluated by the radiologist, showing the difference between the algorithm and the physician’s opinions. The value of R-parameter around 0.97 was obtained for all groups. The expected linear dependency between the radiologist measures and the algorithm was observed. The T-student test did not find statistical differences between any group (*p* > 0.05). Figure 6 compares Bland-Altmann LoA for the three groups. It is clear that LoA tends to increase with the size of the tumor.

Table 1 shows mean and differences obtained by the radiologist and the algorithm. The algorithm measurements presented minimum errors in the group of 3 cm < d ≤ 5 cm diameter. Larger HCCs presented greater necrotic parts inside the tumors, which makes its maximum diameter estimation more difficult by radiologist.

**Table 1:** Difference between radiologist and algorithm estimation of maximum contrasted diameter of HCC

**Group**	**Images**	$\bar{M}\pm 2\times \mathrm{SD}$**(cm)**			**R**^**a**^	***p***^**b**^
		**Radiologist**	**Algorithm**	**Algorithm and radiologist difference**		
d ≤ 3 cm	12	2.2 ± 0.5	2.1 ± 0.6	0.12 ± 0.58	0.78	0.17
3 cm < d ≤ 5 cm	28	4.0 ± 0.6	4.0 ± 0.7	- 0.05 ± 0.86	0.61	0.54
d > 6 cm	23	8.4 ± 2.7	8.2 ± 2.4	0.25 ± 1.14	0.96	0.05

^a^R-Squared correlation coefficients between data and linear fit.

^b^*p* values based on t-test.

Difference between radiologist and algorithm estimation of maximum contrasted diameter of HCC.

Of interest, the algorithm was able to measure area and volume of the tumors evaluated. This can be an important tool contributing to a better assessment of the tumor in the clinical routine.

## Conclusions

Due to the relevance of the tumor size to choose the best treatment for each patient with HCC lesions in the liver, the maximum diameter of the HCC lesions should be considered as a measurement that requires other resent complementary measurement methods. When the tumor maximum diameter is close to the limits established for the OLT inclusion criteria and there was not tumor spread outside the liver, TACE is one of the most treatment modality used to reduce HCC lesions, but the evaluation of contrast-enhanced exams after TACE procedures can be difficult because the lesions tend to be many necrotic areas within the tumor tissue. Since there are strict criteria used to indicate which treatment must be performed to patients with HCC and the most used criteria are based on the tumor size, a non-subjective evaluation is an important tool to be considered in this case.

Currently, the new concept of tumor viable tissue is coming to improve the assessment of HCC lesions, especially after TACE procedures. Some studies showed that mRECIST and EASL criteria are suitable to evaluate these tumors, being now considered more adequate than the previous WHO and RECIST. They are more closely correlated to clinical endpoints [30] and are easier to use. However, they can lead to variations in the evaluations obtained from different radiologists, whom are not able to calculate the viable tumor area.

The analysis of the viable tumor area can be very important after TACE, discriminating the lesions that really respond after the procedure. TACE often lead to coagulative necrotic lesions in the central area of HCC tumors, and it can be difficult to be measured using only the current criteria [30]. For instance, a tumor of 5 cm with a necrotic are of 1 cm in the central area is different of a tumor of the same size with a central necrotic area of 4 cm, but both have the same maximum diameter according to the current criteria, that take into account the longest measure of viable tumor tissue and not the necrotic area. On the other hand, the analysis of the viable tumor area can show the difference in these two tumors after a TACE procedure because it calculates the area of viable tumor in each lesion. Additionally, it can be used to compare the efficacy of TACE procedures using different chemotherapeutic agents or of each hemodynamic service.

Therefore, a single dimension does not take into account the viable area of a given tumor, and better evaluation methods are needed to avoid misinterpretations about each nodule found at the contrast-enhanced exam. In this study, we have presented a novel method to measure contrast-enhanced HCC nodules in liver CT exams. Our method was able to distinguish the normal liver tissue from the cancer, by using wavelets base to determine accurately the tumors’ limits. This image treatment plays an important role in the lesions characterization, properly measuring the size of HCC nodules. The algorithm was able to measure small-sized tumors with great accuracy. The precision is gradually decreasing for large-sized lesions (diameter > 5 cm), but still with enough precision to apply the used treatment criteria.

Our results confirm the findings of previous studies, suggesting that the maximum diameter should not be used alone to represent the tumor size. Many HCC tumors are noncircular lesions and may be only approximated by an ellipse, as described by Jensen et al. [31]. Based on these findings, patients with different tumors’ shapes can be misevaluated if only the maximum diameter would be used to classify the disease according to the mRECIST criteria. Therefore, the HCC tumors evaluation can be improved if the total viable tumor area could be analyzed, especially for tumors submitted to TACE procedures, which often have coagulative necrotic areas inside of them. This kind of evaluation of the local response after TACE could lead to a more detailed comparison between the local response and the clinical end points, but it needs to be evaluated in further studies. The trend to major difference for lesions >5 cm may be associated with necrosis caused by TACE, leading radiologists to overestimate lesions. For the moment, the algorithm presented in our study can be very useful to analyze the viable tumor area without subjective measurements and assist in the final decision clinic.

## Competing interests

The authors declare that they have no competing interests.

## Authors’ contributions

MA carried out the algorithm development and testing, image processing steps, statistical analysis and drafted the manuscript. DRP helped in the design of the study and helped to draft the manuscript. FGR gave the physicians point of view, participated in the design of the study and helped to draft the manuscript. SBD participated in the statistical analysis and helped to draft the manuscript. JRAM conceived the study, participated in its design and coordination and helped to draft the manuscript. All authors read and approved the final manuscript.

## References

[B1] Ferlay J Bray F Pisani P Parkin DM GLOBOCAN 2000: Cancer Incidence, Mortality and Prevalence Worldwide, version 1.0. International Agency for Research on Cancer CancerBase no. 5 2001 Lyon, France: IARC Press

[B2] El-Serag HB Davila JA Surveillance for hepatocellular carcinoma: in whom and how? Ther Adv Gastroenterol 2011 4 5 10 10.1177/1756283X10385964 PMC3036965 21317990

[B3] Llovet JM Bru C Bruix J Prognosis of hepatocellular carcinoma: the BCLC staging classification Semin Liver Dis 1999 19 329 338 10.1055/s-2007-1007122 10518312

[B4] El-Serag HB Hepatocellular carcinoma N Engl J Med 2011 365 1118 1127 10.1056/NEJMra1001683 21992124

[B5] Lau WY Management of hepatocellular carcinoma J R Coll Surg Edinb 2002 47 389 399 11874260

[B6] Mazzaferro V Regalia E Doci R Andreola S Pulvirenti A Bozzetti F Montalto F Ammatuna M Morabito A Gennari L Liver transplantation for the treatment of small hepatocellular carcinomas in patients with cirrhosis N Engl J Med 1996 334 693 699 10.1056/NEJM199603143341104 8594428

[B7] Lencioni R Llovet JM Modified RECIST (mRECIST) assessment for hepatocellular carcinoma Semin Liver Dis 2010 30 52 60 10.1055/s-0030-1247132 20175033 PMC12268942

[B8] Sato Y Watanabe H Sone M Onaya H Sakamoto N Osuga K Takahashi M Arai Y Tumor response evaluation criteria for HCC (hepatocellular carcinoma) treated using TACE (transcatheter arterial chemoembolization): RECIST (response evaluation criteria in solid tumors) version 1.1 and mRECIST (modified RECIST): JIVROSG-0602 Ups J Med Sci 2013 118 16 22 10.3109/03009734.2012.729104 23167460 PMC3572665

[B9] Lin CT Hsu KF Chen TW Yu JC Chan DC Yu CY Hsieh TY Fan HL Kuo SM Chung KP Hsieh CB Comparing hepatic resection and transarterial chemoembolization for Barcelona Clinic Liver Cancer (BCLC) stage B hepatocellular carcinoma: change for treatment of choice? World J Surg 2010 34 2155 2161 10.1007/s00268-010-0598-x 20407768

[B10] Mallat S A wavelet Tour of Signal Processing 1999 New York: Academic Press

[B11] Alvarez M Pina DR Miranda JR Duarte SB Application of wavelets to the evaluation of phantom images for mammography quality control Phys Med Biol 2012 57 7177 7190 10.1088/0031-9155/57/21/7177 23060095

[B12] Mallat S Applied Mathematics meets Signal Processing Challenges for the 21st Century 2000 138 161

[B13] Shidahara M Tsoumpas C McGinnity CJ Kato T Tamura H Hammers A Watabe H Turkheimer FE Wavelet-based resolution recovery using an anatomical prior provides quantitative recovery for human population phantom PET [(1)(1)C]raclopride data Phys Med Biol 2012 57 3107 3122 10.1088/0031-9155/57/10/3107 22547469

[B14] Gonzalez RC Woods RE Digital Image Processing 2002 2 Upper Saddler River, NJ: Prentice Hall

[B15] Bovik AC Handbook of Image and Video Processing 2005 1 San Diego, CA: Elsevier Academic Press

[B16] Daubechies I Ondelettes Science 1993 262 1589 1591 10.1126/science.262.5139.1589 17829387

[B17] Bae KT Giger ML Chen CT Kahn CE Jr Automatic segmentation of liver structure in CT images Med Phys 1993 20 71 78 10.1118/1.597064 8455515

[B18] Markwardt K Wavelet Analysis and Frequency Band Decompositions 2006 (Editor ed.^eds.). City

[B19] Daubechies I Roussos E Takerkart S Benharrosh M Golden C D’Ardenne K Richter W Cohen JD Haxby J Independent component analysis for brain fMRI does not select for independence Proc Natl Acad Sci USA 2009 106 10415 10422 10.1073/pnas.0903525106 19556548 PMC2705604

[B20] Alzubi S Islam N Abbod M Multiresolution analysis using wavelet, ridgelet, and curvelet transforms for medical image segmentation Int J Biomed Imag 2011 2011 136034 10.1155/2011/136034 PMC3173970 21960988

[B21] Chen YT Tseng DC Wavelet-based medical image compression with adaptive prediction Comput Med Imaging Graph 2007 31 1 8 10.1016/j.compmedimag.2006.08.003 17046200

[B22] Dandapat S Xu J Chutatape O Krishnan SM Wavelet transform domain data embedding in a medical image Conf Proc IEEE Eng Med Biol Soc 2004 2 1541 1544 17271991 10.1109/IEMBS.2004.1403471

[B23] Guihong Q Dali Z Pingfan Y Medical image fusion by wavelet transform modulus maxima Opt Express 2001 9 184 190 10.1364/OE.9.000184 19421288

[B24] Hou W Wu X Peng C An algorithm of a wavelet-based medical image quantization Sheng Wu Yi Xue Gong Cheng Xue Za Zhi 2002 19 657 659 675 12561372

[B25] Landin CJ Reyes MM Martin AS Rosas RM Ramirez JL Ponomaryov V Soto MD Medical image processing using novel wavelet filters based on atomic functions: optimal medical image compression Adv Exp Med Biol 2011 696 497 504 10.1007/978-1-4419-7046-6_50 21431590

[B26] Liu H Chen Z Chen X Chen Y Multiresolution medical image segmentation based on wavelet transform Conf Proc IEEE Eng Med Biol Soc 2005 4 3418 3421 17280957 10.1109/IEMBS.2005.1617212

[B27] Korfiatis P Skiadopoulos S Sakellaropoulos P Kalogeropoulou C Costaridou L Combining 2D wavelet edge highlighting and 3D thresholding for lung segmentation in thin-slice CT Br J Radiol 2007 80 996 1004 10.1259/bjr/20861881 18065645

[B28] Gonzalez RC Woods RE Eddins SL Digital Image Processing using MATLAB 2004 Upper Saddle River, N. J: Pearson Prentice Hall

[B29] Bland JM Altman DG Statistical methods for assessing agreement between two methods of clinical measurement Lancet 1986 1 307 310 2868172

[B30] Jung ES Kim JH Yoon EL Lee HJ Lee SJ Suh SJ Lee BJ Seo YS Yim HJ Seo TS Lee CH Yeon JE Park JJ Kim JS Bak YT Byun KS Comparison of the methods for tumor response assessment in patients with hepatocellular carcinoma undergoing transarterial chemoembolization J Hepatol 2013 58 1181 1187 23395691 10.1016/j.jhep.2013.01.039

[B31] Jensen MM Jorgensen JT Binderup T Kjaer A Tumor volume in subcutaneous mouse xenografts measured by microCT is more accurate and reproducible than determined by 18F-FDG-microPET or external caliper BMC Med Imaging 2008 8 16 10.1186/1471-2342-8-16 18925932 PMC2575188

